# Regional variations in trajectories of long-term readmission rates among patients in England with heart failure

**DOI:** 10.1186/s12872-019-1057-8

**Published:** 2019-04-06

**Authors:** Ahsan Rao, Dani Kim, Ara Darzi, Azeem Majeed, Paul Aylin, Alex Bottle

**Affiliations:** 1 Dr Foster Unit, Department of Public Health, Imperial College London, 3 Dorset Rise, London, EC4Y 8EN UK; 2 Department of Surgery, Imperial College London, St Mary’s Hospital, Praed Street, London, W2 1NY UK

**Keywords:** Heart failure, Regional analysis, Readmission rate, High-impact users

## Abstract

**Background:**

We aimed to compare the characteristics and types of heart failure (HF) patients termed “high-impact users”, with high long-term readmission rates, in different regions in England. This will allow clinical factors to be identified in areas with potentially poor quality of care.

**Methods:**

Patients with a primary diagnosis of heart failure (HF) in the period 2008–2009 were identified using nationally representative primary care data linked to national hospital data and followed up for 5 years. Group-based trajectory models and sequence analysis were applied to their readmissions.

**Results:**

In each of the 8 NHS England regions, multiple discrete groups were identified. All the regions had high-impact users. The group with an initially high readmission rate followed by a rapid decline in the rate ranged from 2.5 to 11.3% across the regions. The group with constantly high readmission rate compared with other groups ranged from 1.9 to 12.1%. Covariates that were commonly found to have an association with high-impact users among most of the regions were chronic respiratory disease, chronic renal disease, stroke, anaemia, mood disorder, and cardiac arrhythmia. Respiratory tract infection, urinary infection, cardiopulmonary signs and symptoms and exacerbation of heart failure were common causes in the sequences of readmissions among high-impact users in all regions.

**Conclusion:**

There is regional variation in England in readmission and mortality rates and in the proportions of HF patients who are high-impact users.

## Background

Heart failure (HF) is one of the commonest reasons for hospitalisation in adults [1]. The burden of HF is rising globally because of ageing populations [2]. The Centers for Medicare and Medicaid Services’ (CMS) Hospital Readmission Reduction Program in the US currently penalises hospitals financially for higher than expected risk adjusted 30-day readmission rates for HF [3]. Emergency readmissions are a marker of poor patient health status, decline in quality of life as well as quality of care problems [3]. There is an ongoing discussion about the effectiveness of the 30-day readmission rate as a quality metric as many HF patients have multiple readmissions after 30 days [3]. For the general patient population, only a quarter of 30-day readmissions are potentially avoidable [4]. The concept of “high-impact users”, those with a high readmission rate, e.g. three unplanned admissions within a year, goes beyond the 30-day window [5]. Identification of these patients can help healthcare providers to formulate targeted policies in case-management programmes.

It is equally important to investigate the causes of emergency readmissions to assess if there is a repeated cycle of events that are common in these patients that can be potentially avoided in the community [6]. To avoid regression to the mean, which happens when patients have a crisis and high admission rates followed by a return to baseline rates, we have previously used trajectory modelling of rates over a five-year period [7].

International clinical trials have suggested variation in overall hospitalization and readmission due to exacerbation of HF in different regions of the world [8]. Hospitals in North America were shown to have the highest rate of hospitalizations [8]. Comparison of outcomes in HF patients among different countries showed disparity in prescription of medical therapy, patients undergoing diagnostic investigations, behavioural counselling and placement of implantable devices and other interventions [9]. Some countries have also documented inter-state variation in the outcomes of HF patients [10]; we previously compared admissions for HF in England and its regions with those in Lombardy in northern Italy and found that, despite key similarities in healthcare systems, English and especially London patients of a given age and gender spent on average much more time in hospital for HF but lived longer following their index admission than patients in Lombardy [11].

We investigate whether there is variation in the proportion and characteristics of high-impact users among HF patients by geographical region in England. Such variations could be partly due to differences in the medical management of these patients. Areas with higher proportions of high-impact users can then be identified and local policies implemented to improve outcomes.

## Methods

Data were obtained from Clinical Practice Research Datalink (CPRD) linked to Hospital Episode Statistics (HES) and the Office for National Statistics (ONS) deaths database. CPRD is the largest national primary care database with over 13 million enrolled patient medical records across the country and contains 8.5% of the patient population in England [12]. It includes information on demographics, medical diagnosis and procedural information for each consultation with the general practitioner (GP). The fact and date of death are obtained from ONS (Office for National Statistics). Around 660 practices have volunteered in CRPD to share patient data. Around 60% of the general practices contributing to CPRD are linked to HES data.

Patients over the age of 18 with a first-time diagnosis of heart failure recorded between 1st Apr 2008 and 31st March 2009 were included in the study if they contributed to CPRD and allowed linkage to the other datasets. Medical codes (“medcodes”) from CRPD were used to identify patients who were first documented to have heart failure. Medcodes correspond to Read codes, which are part of the standard terminology system used by general practices in the UK. Also, the ICD-10 (International Classification of Diseases) code I50X was used to identify patients who were diagnosed with HF in hospital*.* All patient records were traced back at least 5 years to verify the absence of any earlier HF diagnosis and also to retrieve data on their past medical history, and social and management-related factors. All information on any hospital admission with its primary diagnosis and mortality was obtained during the study period. In this retrospective cohort study, patients were followed up for five years up to March 2014.

The CPRD data include a field for region in England: North-East, Yorkshire and Humber, North-West, East of England, London, South-East, South-West, South-Central, and West-Midlands. The number of patients was very low in Yorkshire and Humber region; hence, they were grouped together with North-East region.

The assessed risk factors were identified from previous studies and categorised into patient-based, social and lifestyle related, and management-based factors. The patient-based factors consisted of age at diagnosis, sex, and past medical history recorded in the last 5 years preceding the diagnosis of HF. Age was grouped into categories for use in the model in the following brackets: 18–45, 45–54, 55–64, 65–74, 75–84, and 85+ − an adaption from previous studies [13]. The social and lifestyle factors included impact of bereavement, marital or relationship problems, history of smoking and heavy alcohol intake. These factors were chosen because they impact on the prognosis of heart failure [14]. The patients with ex-smoking and current smoking status in the preceding years were identified through medcodes; smoking was categorised as either former or current. Bereavement was defined as loss of an immediate family member.

The management-based factors consisted of GP visit coded for the monitoring of renal function, flu vaccination, measurement of blood pressure and exercise recommendation. Other factors included 3 or more emergency admissions for any reason other than HF in the year preceding the diagnosis of HF, the number of annual GP visits (including out-of-hours) and the number of out-of-hours GP visits in the year preceding the diagnosis of HF. Previous annual GP visits and annual out-of-hours GP visits did not have a linear relationship with the high-impact users. Hence, they were categorised according to percentile ranges (<25th, 26-50th, 51-75th and > 75th percentiles). The effect of HF diagnosed as an inpatient and history of use of medications for treatment of signs and symptoms of HF was also evaluated. Medication use for the management of signs and symptoms of heart failure included prescriptions for drugs like loop and thiazide diuretics, beta-blockers, angiotensin-converting-enzyme inhibitors, angiotensin II receptor antagonists, digoxin, and bumetanide [15]. The information on social factors and management-based factors was obtained for 5 years before the date of diagnosis of HF.

Group-based trajectory modelling (GBTM) was applied to the data to categorise patients into subgroups with different trends in readmission rates. The outcome was the annual number of emergency readmissions for each patient for each successive year during the follow-up period. The patients who died during their first hospital admission where they were diagnosed with HF were excluded from the analysis. They were part of the low-risk group and were used to compare the outcomes with other groups. In order to determine the optimum number of subgroups within a population, the choice of model was based on the following criteria: the smallest value of Bayesian Information Criteria (BIC), largest value for average posterior probability for each group, odds of correct classification (OCC) > 5 and each trajectory with significant parameter estimates (*p* <  0.05). These criteria are usually chosen to test the model with the best estimate of number of groups and predictors associated with them [16–18]. The Statistical Analysis Software (SAS v9.4) was used to apply GBTM on the datasets via the macro ‘Proc Traj’ [19]. For each subgroup within the patient population, the average number of readmissions annually was measured and depicted on the graph. The group showing highest annual readmission rate persistently in the follow up period was termed as high risk group and those with consistently low average readmission rate were classed as low risk group. The group with mediocre annual readmission rate in the follow up period was categorised as intermediate group. Multinomial logistic regression model was used to assess co-variates associated with high-impact users and low-impact group was used a reference for comparison.

Sequence analysis was performed to identify common causes (primary diagnoses) and their pattern of emergency admissions among subgroups of HF population using the *‘TraMineR’* package in R [20]. The primary diagnosis codes were grouped together based on their common pathology.

## Results

### General patient characteristics

There were a total of 10,317 patients and the population were divided into 8 regions: London (*n* = 1175), East of England (*n* = 1249), North-East (*n* = 996), North-West (*n* = 1788), South-Central (*n* = 1307), South-East (*n* = 1238), West-Midlands (*n* = 1189) and South-West (*n* = 1375). The proportion of low-impact patients with minimal readmission rates in each region were as following: London (48.4%), South-East (51%), North-West (51.1%), North-East (64.7%), South-Central (65%), East of England (65.8%), West-Midlands (66.4%), and South-West (76.4%). Patient characteristics by region are shown in Table 1*.* Significant regional variation was seen in their basic demographics and past medical history. The proportion of patients with a history of myocardial infarction was lowest in the South-East, South-Central and South-West regions. The proportion of patients with congenital heart disease was higher in London and the North-West region. The proportion of patients with a background of hypertension and cardiac arrhythmia was higher in the Southern and West Midlands regions, but proportions with renal failure and cardiomyopathy were lower. The number of patients with GP visits for HF medication review and prescription were lower in Northern, East of England and West Midlands regions. The proportion of patients with increased hospital admissions before HF diagnosis was high in East of England, London and North-West region. Out-of-hours GP visits in a year preceding HF diagnosis were highest in East of England, North-West and South-Central region.

**Table 1:** Patient characteristics in general HF population and different regions. The co-morbidities and mortality of each region was compared with the general population

Patient characteristics N [%] or mean [SD]	Overall population	East of England	London	North-East	North-West	South-east	South-west	South-central	West Midlands
Age (mean [SD])	76.2 [14.3]	79.1 [11.9]^	78.6 [11.5]^	74.7 [15.0]**	75.9 [12.5]	79.8 [11.2]^	78.6 [11.4]^	79.6 [11.2]^	76.3 [13.9]
Female sex	3520 [37.2]	404 [32.3]^	317 [26.9]^	484 [48.5]^	530 [29.6]^	395 [31.9]^	386 [28.1]^	380 [29.1]^	624 [52.4]^
Myocardial infarction	1338 [14.1]	177 [14.2]	143 [12.1]	246 [24.7]^	255 [14.2]	151 [12.2]	164 [11.9]	150 [11.5]	250 [21.2]^
Atrial fibrillation	3174 [33.5]	424 [33.9]	327 [27.8]^	479 [48.2]^	509 [28.4]^	423 [34.2]^	382 [27.8]^	392 [29.9]^	626 [53.1]^
Myocarditis/Cardiomyopathy	303 [3.2]	30 [2.4]	46 [3.9]	61 [6.1]^	43 [2.4]	53 [4.3]	36 [2.6]	36 [2.7]	69 [5.8]^
Hypertension	4494 [47.4]	584 [46.7]	517 [44.0]*	742 [74.7]^	790 [44.1]*	567 [45.7]	511 [37.2]^	501 [38.3]^	860 [73.0]^
Diabetes	1570 [16.6]	195 [23.6]	178 [15.1]	250 [25.1]^	293 [16.3]	191 [15.4]	193 [14.0]	177 [13.5]*	274 [23.2]^
Valvular heart disease	1392 [14.7]	162 [12.9]	150 [12.7]	225 [22.6]^	276 [15.4]	181 [14.6]	168 [12.2]	166 [12.7]	270 [22.9]^
Peripheral vascular disease	586 [6.2]	63 [5.0]	74 [6.3]	106 [10.6]^	117 [6.5]	66 [5.3]	63 [4.6]	73 [5.6]	95 [8.0]*
Dementia	908 [9.8]	112 [8.9]	124 [10.5]	160 [16.1]^	145 [8.1]	94 [7.6]*	96 [6.9]	126 [9.6]	185 [15.7]^
HF diagnosed as an inpatient	4795 [50.6]	548 [43.8]^	430 [36.5]^	743 [74.6]^	658 [36.8]^	493 [39.8]^	504 [36.6]^	532 [40.7]^	887 [74.6]^
Overall 5-year mortality	3741 [39.5]	402 [32.2]^	360 [30.6]^	485 [48.6]^	565 [31.5]^	400 [32.3]^	421 [30.6]^	454 [34.7]^	646 [54.3]^

* denotes *P* value < 0.05, ** denotes P value < 0.01 and ^ denotes *P* value < 0.001

### Classification of groups in different regions

In every region, multiple discrete groups were identified where the majority of the patients belonged to a low-impact group with persistently low readmission rates *(*Table 2*).* The group with initially high readmission rates followed by a rapid decline in their rate was labelled as the short-term high-impact group; the proportion of these patients ranged from 2.5 to 11.3%. The group with constantly high readmission rates was labelled as the chronic high-impact group and was present in all regions; the proportion of patients in this group varied from 1.9 to 12.1%. The remaining groups with moderate readmission rates were classed as intermediate groups. North-East region had the highest mortality rate and the highest short-term impact users, whereas, North-West region had lower mortality and higher proportion of chronic high-impact users. In most regions, the number of intermediate groups was 2 except London and South-West region where there was only 1 intermediate group; the proportion of patients in this group was 11.5 to 40.1%.

**Table 2:** The modelling of HF patients into different groups in each region. (BIC: Bayesian Information criterion)

Regions	Total No. of patients	No. of groups	Intermediate groups (n)	Proportion of intermediate users	High-impact groups (n)	Types of high-impact users	Proportion of high-impact users
London	1175	4	1	38.8%	2	Short-term	6.6%
						Chronic	6.2%
East of England	1249	5	2	29.5%	2	Short-term	2.5%
						Chronic	2.2%
North-East	996	5	2	21.9%	2	Short-term	11.3%
						Chronic	2.1%
North-West	1788	4	1	33.8%	2	Short-term	4.9%
						Chronic	10.2%
South-Central	1307	5	2	25.9%	2	Short-term	7.2%
						Chronic	1.9%
South-East	1238	5	2	40.1%	2	Short-term	6.2%
						Chronic	2.7%
West-Midlands	1189	5	2	23.4%	2	Short-term	8.6%
						Chronic	1.6%
South-West	1375	3	1	11.5%	1	Chronic	12.1%

**Fig. 1:**
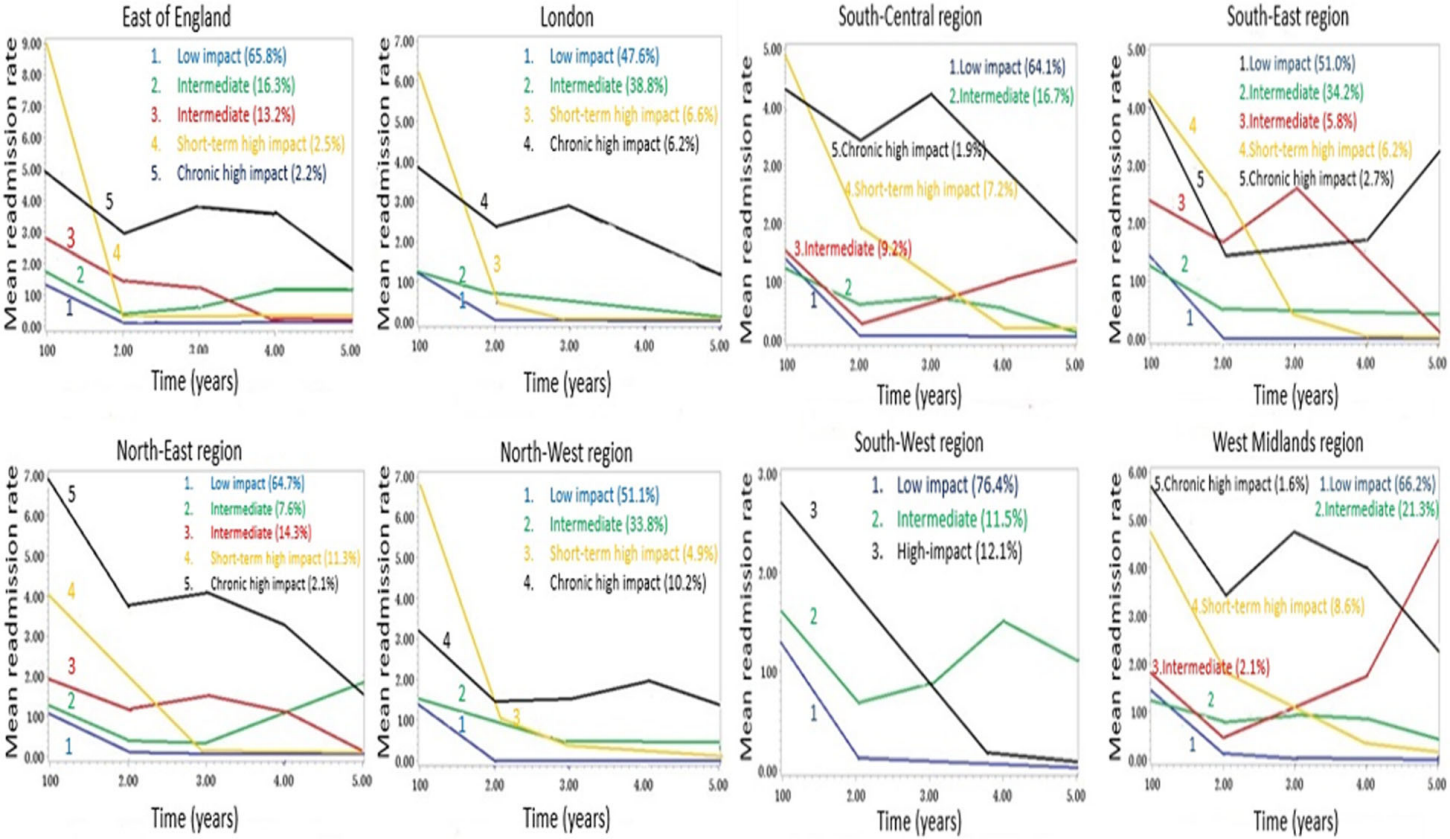
Trajectory of subgroups in different regions of England based on mean readmission rate

The pattern of change of readmission rate of each group in different regions over the follow-up period is shown in Fig. 1a and b*.* The initial mean readmission rate among short-term high-impact users was the highest, between 6 and 9, in the East of England, London and South-West regions. The initial mean readmission rate among chronic high-impact users was usually between 4 and 7, which gradually declined over time except in the South-East region, where it rose later. Of the regions with 2 intermediate groups, one intermediate group had a gradual decline in the readmission rate, while the readmission rate either remained constant or moderately increased in the other group. In the West-Midlands region, there was a sharp rise in the readmission rate among patients in the intermediate group. Of all the regions, the South-West region had the lowest mean readmission rate, and it remained lowest throughout the following years.

### Covariates associated with high-impact users

The covariates that were commonly found to have an association with short-term high-impact users among most of the regions were chronic respiratory disease, chronic renal disease, stroke, anaemia, mood disorder, and cardiac arrhythmia (Table 3). Older age patients had lower odds of being associated with short-term high-impact users. Similar covariates were found to be associated with chronic high-impact users. Among the intermediate users from all regions, hypertension was the prominent predictor followed by atrial fibrillation and chronic renal disease *(*Table 4*).* Diagnosis of HF as an inpatient and history of GP visit for review of HF medications had lower odds of being associated with the intermediate group.

**Table 3:** Co-variates associated with short-term and chronic high-impact groups in different regions

Short-term high-impact	OR [95% CI]	Chronic high-impact	OR [95% CI]
London			
Stroke^	4.39 [2.51–7.69]	Chronic renal disease+	11.82 [6.05–23.10]
Anaemia^	4.26 [2.56–7.10]	Diabetes+	8.76 [5.05–15.18]
Chronic renal disease*	4.01 [2.23–7.24]	Valvular heart disease+	5.81 [3.39–9.97]
Valvular heart disease*	3.06 [1.84–5.10]	Stroke+	5.64 [3.16–10.07]
Chronic respiratory disease*	2.86 [1.75–4.66]	Chronic respiratory disease+	4.62 [2.64–8.08]
		Female sex*	2.89 [1.68–4.95]
		Anaemia*	2.75 [1.65–4.57]
East of England			
Anaemia*	8.33 [3.56–19.49]		
North-East			
Congenital heart disease*	3.74 [2.27–6.17]	Hypertension+	14.88 [8.00–27.66]
Chronic renal disease+	3.00 [2.64–3.42]	Cardiac arrhythmia+	6.17 [5.00–7.61]
Dementia+	2.86 [2.46–3.32]	Mood disorder+	5.81 [4.62–7.32]
Cardiac arrhythmia+	2.75 [2.41–3.13]	Chronic respiratory disease+	5.31 [4.18–6.75]
Hypertension+	2.59 [2.16–3.10]	Chronic renal disease+	4.90 [3.97–6.05]
Mood disorder+	2.51 [2.12–2.97]	Anaemia+	4.14 [3.39–5.05]
Chronic respiratory disease+	2.32 [2.03–2.64]	Dementia+	3.86 [2.97–5.00]
Anaemia+	2.23 [1.95–2.53]	Pulmonary embolism^	2.66 [1.86–3.82]
Valvular heart disease+	2.12 [1.84–2.44]	Diabetes+	2.44 [1.99–2.97]
Stroke+	1.65 [1.49–1.82]	Stroke+	2.29 [1.88–2.80]
Atrial fibrillation*	1.35 [1.22–1.49]	Atrial fibrillation+	2.18 [1.75–2.72]
Older age+	0.74 [0.69–0.79]	Marital problems*	2.18 [1.52–3.13]
HF diagnosis as an inpatient^	0.61 [0.51–0.72]	Valvular heart disease^	2.12 [1.73–2.59]
		Peripheral vascular disease*	1.82 [1.43–2.32]
		Ischaemic heart disease*	1.58 [1.28–1.95]
		Exercise recommendation by GP*	1.55 [1.25–1.93]
		History of flu vaccination+	1.52 [1.25–1.86]
		Older age+	0.41 [0.37–0.46]
North-West			
Hypertension+	5.64 [3.42–9.30]	Older age*	0.43 [0.31–0.59]
Mood disorder+	3.29 [2.32–4.66]	Number of patients with increased out-of-hours GP visits (>90th percentile) ^	0.34 [0.23–0.50]
Dementia^	3.13 [2.16–4.53]	GP visit for renal function monitoring*	0.31 [0.19–0.51]
Cardiac arrhythmia^	2.83 [2.10–3.82]	HF diagnosis as an inpatient+	0.19 [0.13–0.28]
Chronic renal disease^	2.46 [1.82–3.32]		
Anaemia^	2.39 [1.77–3.22]		
Chronic respiratory disease*	2.20 [1.63–2.97]		
History of flu vaccination*	2.12 [1.54–2.92]		
Older age*	0.48 [0.33–0.68]		
HF diagnosis as an inpatient*	0.41 [0.27–0.61]		
South-Central			
Chronic respiratory disease^	5.93 [3.63–9.68]	Hypertension^	0.31 [0.17–0.55]
Valvular heart disease+	5.75 [3.49–9.49]	Chronic renal disease*	0.22 [0.14–0.37]
Cardiac arrhythmia^	4.62 [2.64–8.08]	Older age*	0.23 [0.12–0.41]
Older age^	0.22 [0.13–0.39]	Dementia*	0.19 [0.10–0.37]
		HF diagnosis as an inpatient+	0.06 [0.03–0.11]
South-East			
Ischaemic heart disease+	17.64 [4.76–65.37]	Chronic renal disease*	4.35 [3.00–6.30]
Chronic renal disease^	8.76 [4.95–15.49]	Anaemia+	4.14 [2.89–5.93]
Chronic respiratory disease^	5.53 [3.06–9.97]	Dementia^	3.16 [2.05–4.85]
Cardiac arrhythmia*	5.16 [3.00–8.85]	Cardiac arrhythmia*	2.61 [1.80–3.78]
		Chronic respiratory disease+	2.23 [1.55–3.19]
		GP visit for HF medication review*	0.38 [0.24–0.62]
		Older age*	0.37 [0.23–0.58]
West-Midlands			
Cardiac arrhythmia+	5.47 [3.49–8.58]	Chronic renal disease+	9.97 [5.21–19.11]
Hypertension*	4.06 [2.10–7.85]	Cardiac arrhythmia*	5.70 [3.22–10.07]
Chronic respiratory disease^	3.32 [2.18–5.05]	Stroke^	5.16 [2.94–9.03]
Female sex*	2.75 [1.73–4.35]	Chronic respiratory disease^	4.62 [2.61–8.17]
Older age*	0.39 [0.25–0.63]	Older age*	0.26 [0.15–0.47]
South-West			
		History of flu vaccination*	9.03 [3.00–27.11]
		Chronic respiratory disease*	0.07 [0.02–0.25]
		Older age*	0.03 [0.01–0.11]
		HF diagnosis as an inpatient+	0.01 [0.00–0.04]

(* denotes P <  0.05, ^ denotes P <  0.01, + denotes P <  0.001). Age was grouped into categories for use in the model in the following brackets: 18–45, 45–54, 55–64, 65–74, 75–84, and 85 +)

**Table 4:** Significant co-variates associated with intermediate groups in different regions

Intermediate group	OR [95% CI]	P value
London		
Hypertension	2.80 [1.95–4.01]	0.003
Chronic renal disease	1.65 [1.27–2.14]	0.05
Chronic respiratory disease	1.63 [1.27–2.10]	0.05
Number of patients with previous GP visit for HF medication review	0.51 [0.37–0.71]	0.04
Older age	0.35 [0.26–0.46]	0.002
East of England		
Diabetes	0.32 [0.18–0.54]	0.03
Chronic respiratory disease	0.32 [0.19–0.54]	0.03
Chronic renal disease	0.21 [0.12–0.37]	0.005
North-East		
Cardiac congenital conditions	4.31 [2.51–7.39]	0.007
Hypertension	3.90 [3.32–4.57]	< 0.001
Ischaemic heart disease	2.36 [1.79–3.13]	0.002
Mood disorders	2.32 [2.01–2.66]	< 0.001
Chronic renal diseases	2.03 [1.84–2.25]	< 0.001
Valvular heart disease	1.95 [1.73–2.20]	< 0.001
Cardiac arrhythmias	1.93 [1.75–2.14]	< 0.001
Dementia	1.80 [1.57–2.08]	< 0.001
Respiratory conditions	1.54 [1.39–1.70]	< 0.001
Anaemia	1.54 [1.38–1.72]	< 0.001
Stroke	1.35 [1.20–1.52]	0.012
Atrial fibrillation	1.31 [1.19–1.45]	0.0099
History of smoking	0.87 [0.81–0.93]	0.048
Number of patients with increased out-of-hours GP visits (>90th percentile)	0.64 [0.55–0.75]	0.0028
GP visit for renal monitoring	0.62 [0.53–0.72]	0.0016
Number of patients with previous GP visit for HF medication review	0.62 [0.51–0.76]	0.017
Older age	0.57 [0.53–0.60]	< 0.001
> 3 hospital admissions in preceding year of diagnosis of HF vs. <=2)	0.34 [0.25–0.45]	< 0.001
HF diagnosis as an inpatient	0.32 [0.28–0.37]	< 0.001
North-West		
Hypertension	11.36 [5.53–23.34]	< 0.001
Chronic renal disease	5.81 [4.10–8.25]	< 0.001
Cardiac arrhythmias	4.26 [3.10–5.87]	< 0.001
Atrial fibrillation	2.72 [1.95–3.78]	0.003
Mood disorder	2.61 [1.79–3.82]	0.012
Number of patients with GP visit for flu vaccination	2.51 [1.80–3.49]	0.005
Anaemia	1.97 [1.46–2.66]	0.033
Presentation of atypical signs and symptoms before the diagnosis of HF	0.42 [0.28–0.64]	0.04
HF diagnosis as an inpatient	0.25 [0.16–0.38]	0.001
Older age	0.19 [0.13–0.27]	< 0.001
South-Central		
Chronic respiratory disease	0.36 [0.23–0.56]	0.024
Cardiac arrhythmia	0.25 [0.15–0.43]	0.011
Dementia	0.24 [0.14–0.44]	0.016
Hypertension	0.22 [0.13–0.37]	0.004
Chronic renal disease	0.17 [0.11–0.29]	< 0.001
South-East		
Hypertension	5.37 [2.97–9.68]	0.004
Older age	0.23 [0.16–0.35]	< 0.001
HF diagnosis as an inpatient	0.15 [0.11–0.21]	< 0.001
West-Midlands		
Hypertension	3.16 [2.25–4.44]	< 0.001
Chronic respiratory disease	2.01 [1.55–2.61]	0.007
Atrial fibrillation	1.86 [1.42–2.44]	0.023
Valvular heart disease	1.82 [1.36–2.44]	0.042
HF diagnosis as an inpatient	0.42 [0.32–0.57]	0.003
Older age	0.36 [0.27–0.48]	< 0.001
South-West		
Hypertension	4.81 [3.39–6.82]	< 0.001
Atrial fibrillation	1.80 [1.38–2.36]	0.03
HF diagnosis as an inpatient	0.39 [0.27–0.56]	0.01
Number of patients with previous GP visit for HF medication review	0.28 [0.19–0.41]	< 0.001

### Sequence analysis of causes of emergency readmissions

The commonest causes (primary diagnoses) of emergency admissions were similar in all regions. The top 5 causes of hospital admissions were heart failure, respiratory tract infection, myocardial infarction, atrial fibrillation and external injuries. Respiratory tract infection, urinary infection, cardiopulmonary signs and symptoms and exacerbation of heart failure were common causes in the sequences of readmissions for high-impact users in all regions *(*Table 5*)*. The North-East and North-West regions also had cancer as one of the common causes among the sequences of readmissions. The South-Central and South-East regions had a common occurrence of external injuries in the sequences of readmissions. No common sequences of readmissions were identified among high-impact users in West-Midlands and South-West regions.

**Table 5:** Sequences commonly found among high-impact users as compared with other groups (*P* < 0.001, [UTI, urinary tract infection; RTI, respiratory tract infection; Chest s/s, cardiopulmonary signs and symptoms; Abdo s/s, abdominal signs and symptoms; INJ, external injuries; SALT, speech and swallowing disorders; HF, heart failure]). Other regions did not have particular sequences of readmissions identified

Sequences of readmissions	Low-impact [%]	Intermediate [%]	Short-term high-impact [%]	Chronic high-impact [%]
London				
UTI-RTI	0.4	1.9	4.4	17.5
Chest s/s-Abdo s/s	0.01	4.7	4.4	7.5
RTI-HF	0.4	1.8	8.8	10.0
RTI-UTI	0.4	3.3	8.8	10.0
RTI-Chest s/s	0.01	1.4	4.4	10.0
East of England				
IHD-chest s/s	6.0	0.3	1.9	17.3
RTI-Chest s/s	3.0	0.3	1.9	17.4
Chest s/s-IHD	0.9	0.01	0.9	13.0
Chest s/s-RTI	3.0	0.3	8.8	17.4
MI-IHD	6.0	0.3	6.8	4.3
North-East				
HF-Chest s/s	10.4	3.1	0.9	15.4
UTI-RTI	6.8	0.8	0.6	11.5
Chest s/s-HF	6.5	5.5	0.6	11.5
RTI-Cancer	3.4	4.7	0.01	3.8
North-West				
Chest s/s-RTI	1.2	9.0	9.6	1.1
RTI-INJ	0.3	6.5	2.4	0.4
RTI-cancer	0.6	1.6	4.8	0.01
RT-Chest s/s	0.6	4.1	5.6	0.6
RTI-IHD	0.3	2.4	0.01	4.0
South-Central				
INJ-RTI	2.1	0.01	0.01	12.5
Chest s/s-RTI	0.01	0.01	1.6	6.3
INJ-INJ-RTI	2.1	0.01	0.01	6.3
RTI-INJ	0.7	0.01	0.8	6.3
HF-Chest s/s	1.4	0.01	2.3	6.3
South-East				
UTI-RTI	0.3	0.7	14.3	5.6
RTI-INJ	0.0	3.4	5.7	5.6
INJ-RTI	1.2	2.7	14.3	3.4
INJ-SALT	0.3	1.3	5.7	5.6
SALT-INJ	0.6	0.9	2.8	6.7

## Discussion

Regional analysis of heart failure patients showed that all 8 English regions had more than two subgroups based on annual readmission rates during 5 years’ follow-up. The high-impact users usually had two subgroups: chronic high-impact with constantly high readmission rates and short-term high-impact with initial high readmission rates followed by a rapid immediate decline. A significant number of patients were intermediate users, some of whom had the potential of becoming high-impact users. Risk factors such as chronic respiratory disease, chronic renal disease, stroke, anaemia, mood disorder, and cardiac arrhythmia had a common association with high-impact users in most of the regions. A high proportion of patients among the high-impact group had multiple readmissions, with similar repeated common causes consisting of respiratory tract infection, urine infection, exacerbation of heart failure, ischaemic heart disease and external injuries.

The patients who died during the follow up period were included in the study to provide a pragmatic picture of the observational data following heart failure diagnosis. Short-term high impact users had very high readmission rate in the first year following heart failure and then rapid decline in the readmissions. These patients were also shown to have severe cardio-pulmonary past medical history. Hence, the decline in their readmission rate could have been the result of increased mortality among them after first year following the diagnosis of heart failure.

The proportion of high-impact users varied from 4 to 15% by region: the proportion of high-impact users and their annual readmission rate was lower in southern regions. There was large variation in the characteristics of the patients and the use of healthcare resources among different regions. GP visits for all types of HF medication review and prescriptions were lower in Northern, West Midlands and East of England regions. These regions had a higher number of patients with hospital admissions and out-of-hours GP visits preceding the diagnosis of HF. The recent national audit on hospitals in the UK showed marked variation in the discharge outcomes and in the proportion of patients with HF medication prescription, follow-up imaging, discharge planning and referral to a specialist nurse, cardiologist and cardiac rehabilitation [21].

In every region, intermediate users were a sizable group with the potential to become high-impact users. In each region, they were relatively young population and had similar risk factors. They were more likely than other groups to have hypertension, which was suggestive of the fact that they were prone to get further cardiovascular complications [22, 23]. They also had smaller odds of being associated with regular GP visits for HF medications [24, 25]. It may be the case that these patients complied poorly with their medication, resulting in vascular morbidities in the future [22, 24]. We need to explore other factors that prevent them from becoming high-impact users. It is possible that they may have less severe heart failure or the cause of heart failure is different from high-impact users. In any case, a combination of clinical data with administrative data can help answer these questions.

This study is an initial step towards the demonstration of regional variation in the readmission pathways using epidemiological data. Most of the previous data on regional variation is obtained from multi-centre clinical trials that have assessed the clinical effect of variation in the use of medical therapy for the treatment of heart failure [26, 27]. For most studies, the primary outcome has been the overall mortality and readmission for exacerbation of heart failure condition, whereas some studies have evaluated the overall readmission rate as a secondary measure [8, 27].

Despite variation in co-morbidities of the patients in different regions, the common causes of hospital admissions and their sequences were similar among high-impact patients with multiple hospital readmissions. These patients underwent a vicious cycle of admissions for cardiopulmonary signs and symptoms, chest and urine infections, ischaemic heart disease and external injuries, including falls. Exacerbation of HF is one of the common causes of readmissions. However, these patients get admitted to hospital for other reasons as well. Multiple causes of hospital admissions among HF patients show that the medical management of HF alone cannot yield better outcomes for them [1, 28]. Reducing the readmission rate will require improvement of holistic care of these patients, such as the promotion of secondary preventative measures for ischaemic heart disease, regular flu vaccination, exercise recommendation and prevention of falls and fractures [1, 28–30].

It is important to recognise chronic high-impact users early and assess the option of different care pathways to allow easy transition of care. The predictive model could be used to identify potential high-impact users among heart failure patients by the clinical team. These patients may benefit from closer surveillance. For example, previous work suggests that more aggressive pre- or post-operative cardiopulmonary work-up or rehabilitation to avoid iatrogenic complications may be of benefit following AAA repair. An early and aggressive mobility and cardiopulmonary rehabilitation program for patients in ITU and colonic surgery patients has already been shown to reduce readmission rates [31, 32].

Understanding the structure and provisions of primary care setting in each region may play an important role in understanding regional variation in the trends of readmission rates. The variability in availability and involvement of specialist HF community nurses also need to be assessed in each region. GPs may also differ in their referral rates to specialist community nurses. If they are too late, then the condition may reach at uncompromised severity and it may become hard to prevent exacerbation of the condition. With the current training system, not all GPs spend time on specialist cardiology wards and may not receive hands-on training in the management of heart failure. As a consultant, they may be hesitant in treating the condition and would have low threshold for referral of these patients, as an elective or emergency setting, to specialist care [33]. Furthermore, GP practices have increased trends in recruiting GP with specialist interest. These GPs get additional training in the specialised area, such as cardiology, gynaecology, etc. The proportion of GPs for a population sample in each region and, among those, the proportion of GPs with specialist interest in cardiology is still to be evaluated in each region [34].

This study had certain limitations which need to be considered. Firstly, the analysis did not take into account use of other healthcare resources like outpatient visits or emergency department visits. The categorisation of groups was based on emergency hospital readmission rates alone to understand long-term morbidity and real-life events that impact quality of life. We also focused on emergency hospital admissions because they are a major contributor to health cost among high-impact users. The comparison of the study results with previous studies was limited because very few studies have conducted trajectory and sequence analysis to study long term hospital care use [35]. Secondly, despite coding errors, the Read and ICD coding of the conditions used to select patient cohort have high specificity, and primary diagnoses in HES data are accurate [36, 37]. We tried to use all possible codes that define the condition to include most cases. Thirdly, the use of primary care data linked to hospital data in CPRD does not include all patients suffering from the condition in focus. Six hundred seventy four practices in the UK are registered with CPRD and provide information for primary care data [12, 38]. Approximately half of these practices meet the quality criteria for data input [15]. Not all GP practices are linked with secondary data. However, it is the best available linked data in the country and indeed the largest such database in the world. It is much used for research and provides a great opportunity to assess various hospital- and primary care-based factors and to evaluate long-term outcomes [39]. Fourthly, the regional codes in the CPRD data can only demarcate the country in the broader regions as shown in the study. These regions consist of heterogeneous populations of patients which can present with intra-regional variation in the use of healthcare resources. Further studies are required to investigate differences in healthcare use at a smaller regional distribution and assessing other potentially contributory factors, such as, ethnicity, social support, socio-economic deprivation. This study provides an initial assessment and overview of the differences in the healthcare among regions.

## Conclusions

We found regional variations in five-year rates of readmission and death and in the proportion of high-impact users in HF patients. Potential reasons include quality of care provided at primary and secondary care level. High-impact users comprise a minority but require special support in the community, and further studies are required to assess the factors associated with GP management of the condition.

## Abbreviations

BIC: Bayesian information criteria
CMS: Centre for Medicare and Medicaid services
CPRD: Clinical practice research datalink
GBTM: Group based trajectory modelling
GP: General practitioner
HES: Hospital episode statistics
HF: Heart failure
ICD: International classification of diseases
OCC: Odds of correct classification
ONS: Office of National Statistics

## References

[1] Ziaeian B, Fonarow GC (2016). Epidemiology and aetiology of heart failure. Nat Rev Cardiol.

[2] Abubakar I, Tillmann T, Banerjee A (2015). Global, regional, and national age-sex specific all-cause and cause-specific mortality for 240 causes of death, 1990-2013: a systematic analysis for the global burden of disease study 2013. Lancet.

[3] Dunlay SM, Redfield MM, Weston SA, Long KH, Shah ND, Roger VL (2009). Hospitalizations after heart failure diagnosis. J Am Coll Cardiol.

[4] van Walraven C, Bennett C, Jennings A, Austin PC, Forster AJ (2011). Proportion of hospital readmissions deemed avoidable: a systematic review. CMAJ.

[5] Billings John, Blunt Ian, Steventon Adam, Georghiou Theo, Lewis Geraint, Bardsley Martin (2012). Development of a predictive model to identify inpatients at risk of re-admission within 30 days of discharge (PARR-30). BMJ Open.

[6] Bottle A, Aylin P, Majeed A (2006). Identifying patients at high risk of emergency hospital admissions: a logistic regression analysis. J R Soc Med.

[7] Rao A, Kim D, Darzi A, Majeed A, Aylin P, Bottle A. Long-term trends of use of health service among heart failure patients. Eur Heart J-Quality of Care and Clinical Outcomes. 2018. 10.1093/ehjqcco/qcy013 29718210

[8] Greene SJ, Fonarow GC, Solomon SD, Subacius H, Maggioni AP, Bohm M, Lewis EF, Zannad F, Gheorghiade M (2015). Global variation in clinical profile, management, and post-discharge outcomes among patients hospitalized for worsening chronic heart failure: findings from the ASTRONAUT trial. Eur J Heart Fail.

[9] Mentz RJ, Cotter G, Cleland JG, Stevens SR, Chiswell K, Davison BA, Teerlink JR, Metra M, Voors AA, Grinfeld L, Ruda M, Mareev V, Lotan C, Bloomfield DM, Fiuzat M, Divertz M, Ponikowski P, Massie BM, O'Connor DM (2014). International differences in clinical characteristics, management, and outcomes in acute heart failure patients: better short-term outcomes in patients enrolled in eastern europe and Russia in the PROTECT trial. Eur J Heart Fail.

[10] Lee DS, Johansen H, Gong Y, Hall RE, Cox JL (2004). Regional outcomes of heart failure in Canada. Can J Cardiol.

[11] Bottle A, Ventura CM, Dharmarajan K, Aylin P, Ieva F, Paganoni AM. Regional variation in hospitalisation and mortality in heart failure: comparison of England and lombardy using multistate modelling. Health Care Manag Sci. 2017:1–13. 10.1007/s10729-017-9410-x 28755175

[12] Clinical Practice Research Datalink. CPRD linked data. https://www.cprd.com/dataAccess/linkeddata.asp. Updated 2017. Accessed 04 Feb 2017.

[13] Krumholz HM, Chen Y, Wang Y, Vaccarino V, Radford MJ, Horwitz RI (2000). Predictors of readmission among elderly survivors of admission with heart failure. Am Heart J.

[14] Calvillo–King L, Arnold D, Eubank KJ, Lo M, Yunyongying P, Stieglitz H, Halm EA (2013). Impact of social factors on risk of readmission or mortality in pneumonia and heart failure: systematic review. J Gen Intern Med.

[15] Stocks SJ, Kontopantelis E, Akbarov A, Rodgers S, Avery AJ, Ashcroft DM (2015). Examining variations in prescribing safety in UK general practice: cross sectional study using the clinical practice research datalink. BMJ..

[16] Futoma J, Morris J, Lucas J (2015). A comparison of models for predicting early hospital readmissions. J Biomed Inform.

[17] Sternberg SA, Bentur N, Abrams C, Spalter T, Karpati T, Lemberger J, Heymann AD. Identifying frail older people using predictive modeling. Am J Manag Care. 2012;18(10). 23145847

[18] Nagin D. Group-based modeling of development: Harvard University Press; 2005.

[19] Jones B. Traj: Group-based modelling of longitudinal data. https://www.andrew.cmu.edu/user/bjones/download.htm. Updated 2017. Accessed Dec. 2015.

[20] Gabadinho A, Ritschard G, Studer M, Müller NS (2009). Mining sequence data in R with the TraMineR package: a users guide for version 1.2.

[21] Cleland J, Dargie H, Hardman S, McDonagh T, Mitchell P. National heart failure audit, april 2012-march 2013: NICOR (National Institute for Cardiovascular Outcomes Research) and The British Society for Heart Failure (BSH); 2013.

[22] Kannel WB (1996). Blood pressure as a cardiovascular risk factor: prevention and treatment. JAMA.

[23] Hubert HB, Feinleib M, McNamara PM, Castelli WP (1983). Obesity as an independent risk factor for cardiovascular disease: a 26-year follow-up of participants in the Framingham heart study. Circulation.

[24] McAlister FA, Lawson FM, Teo KK, Armstrong PW (2001). A systematic review of randomized trials of disease management programs in heart failure. Am J Med.

[25] Joffres M, Falaschetti E, Gillespie C, Robitaille C, Loustalot F, Poulter N, McAlister FA, Johansen H, Baclic O, Campbell N (2013). Hypertension prevalence, awareness, treatment and control in national surveys from England, the USA and Canada, and correlation with stroke and ischaemic heart disease mortality: a cross-sectional study. BMJ Open.

[26] Blair JE, Zannad F, Konstam MA, Cook T, Traver B, Burnett J, Grinfeld L, Krasa H, Maggioni AP, Orlandi C, Swedberg K, Udelson JE, Zimmer C, Gheorghiade M (2008). Continental differences in clinical characteristics, management, and outcomes in patients hospitalized with worsening heart failure: results from the EVEREST (efficacy of vasopressin antagonism in heart failure: outcome study with tolvaptan) program. J Am Coll Cardiol.

[27] Pfeffer MA, Claggett B, Assmann SF, boineau B, Anand KS, Clausell N, Desai AS, Diaz R, Fleg JL, gordeev I, Heitner J, Lewis EF, O'Meara E, Rouleau JL, Probsfield JL, Shaburishvili T, Shah SJ, Solomon SD, Sweitzer N, McKinlay S, Pitt B (2015). Regional variation in patients and outcomes in the treatment of preserved cardiac function heart failure with an aldosterone antagonist (TOPCAT) trial. Circulation.

[28] Dickstein K, Cohen-Solal A, Filippatos G, McMurray J, Ponikowski P, Poole-Wilson PA, Stromberg A, van Beldhuisen D, Hoes DAAW, Keren A, Mebazaa A, Nieminen M, Piori SG, Swedberg K (2008). ESC guidelines for the diagnosis and treatment of acute and chronic heart failure 2008. Eur J Heart Fail.

[29] Piepoli MF, Conraads V, Corra U, Dickstein K, Francis D, Jaarsma T, McMurray J, Pieske B, Piotrowicz E, Schmid JP, Anker SD, Solal AC, Filippatos GS, Hoes AW, Gielen S, Giannuzzi P, Ponikowski PP (2011). Exercise training in heart failure: from theory to practice. A consensus document of the heart failure association and the european association for cardiovascular prevention and rehabilitation. Eur J Heart Fail.

[30] Mancia G, De Backer G, Dominiczak A, Cifkova R, Fagard R, Germano G, Grassi G, heagerty AM, Kjeldsen SE, Narkiewicz LK, Ruilope L, Rynkiewicz A, Schmieder RE, Boudier H, Zanchetti A (2007). 2007 guidelines for the management of arterial hypertension: the task force for the management of arterial hypertension of the european society of hypertension (ESH) and of the european society of cardiology (ESC). Eur Heart J.

[31] Basse L, Thorbøl JE, Løssl K, Kehlet H (2004). Colonic surgery with accelerated rehabilitation or conventional care. Dis Colon Rectum.

[32] Suaya JA, Shepard DS, Normand SL, Ades PA, Prottas J, Stason WB (2007). Use of cardiac rehabilitation by medicare beneficiaries after myocardial infarction or coronary bypass surgery. Circulation.

[33] Evans J, Lambert T, Goldacre M. GP recruitment and retention: a qualitative analysis of doctors' comments about training for and working in general practice. Occas Pap R Coll Gen Pract. 2002; (83):iii-vi, 1-33. PMC2560444 12049026

[34] Jones R, Bartholomew J (2002). General practitioners with special clinical interests: a cross-sectional survey. Br J Gen Pract.

[35] Bravata DM, Ho S, Meehan TP, Brass LM, Concato J (2007). Readmission and death after hospitalization for acute ischemic stroke - 5-year follow-up in the medicare population. Stroke.

[36] O'malley KJ, Cook KF, Price MD, Wildes KR, Hurdle JF, Ashton CM (2005). Measuring diagnoses: ICD code accuracy. Health Serv Res.

[37] Dharmarajan K, Hsieh AF, Lin Z, Bueno H, Ross J, Horwitz LI, Barrto-Filho JA, Kim N, Bernheim SM, Suter LG, Drye E, Krumholz HM (2013). Diagnoses and timing of 30-day readmissions after hospitalization for heart failure, acute myocardial infarction, or pneumonia. JAMA.

[38] Herrett E, Gallagher AM, Bhaskaran K, Forbes H, Mathur R, van Staa T, Smeeth L (2015). Data resource profile: clinical practice research datalink (CPRD). Int J Epidemiol.

[39] Chaudhry Z, Mannan F, Gibson-White A, Syed U, Ahmed S, Kousoulis A, Majeed A (2017). Outputs and growth of primary care databases in the United Kingdom: bibliometric analysis. Journal of innovation in health informatics.

